# Habitat Heterogeneity Affects Plant and Arthropod Species Diversity and Turnover in Traditional Cornfields

**DOI:** 10.1371/journal.pone.0128950

**Published:** 2015-07-21

**Authors:** Eliana Martínez, Matthias Rös, María Argenis Bonilla, Rodolfo Dirzo

**Affiliations:** 1 Escuela de Posgrados, Facultad de Ciencias Agrarias, Universidad Nacional de Colombia, Bogotá, Colombia; 2 Instituto de Ecología A. C., Red de Ecoetología, Xalapa, México; 3 Centro Interdisciplinario de Investigación para el Desarrollo Integral Regional, Instituto Politécnico Nacional, Oaxaca, México; 4 Departamento de Biología, Facultad de Ciencias, Universidad Nacional de Colombia, Bogotá, Colombia; 5 Department of Biology, Stanford University, Palo Alto, California, United States of America; University of California, Berkeley, UNITED STATES

## Abstract

The expansion of the agricultural frontier by the clearing of remnant forests has led to human-dominated landscape mosaics. Previous studies have evaluated the effect of these landscape mosaics on arthropod diversity at local spatial scales in temperate and tropical regions, but little is known about fragmentation effects in crop systems, such as the complex tropical traditional crop systems that maintain a high diversity of weeds and arthropods in low-Andean regions. To understand the factors that influence patterns of diversity in human-dominated landscapes, we investigate the effect of land use types on plant and arthropod diversity in traditionally managed cornfields, via surveys of plants and arthropods in twelve traditional cornfields in the Colombian Andes. We estimated alpha and beta diversity to analyze changes in diversity related to land uses within a radius of 100 m to 1 km around each cornfield. We observed that forests influenced alpha diversity of plants, but not of arthropods. Agricultural lands had a positive relationship with plants and herbivores, but a negative relationship with predators. Pastures positively influenced the diversity of plants and arthropods. In addition, forest cover seemed to influence changes in plant species composition and species turnover of herbivore communities among cornfields. The dominant plant species varied among fields, resulting in high differentiation of plant communities. Predator communities also exhibited high turnover among cornfields, but differences in composition arose mainly among rare species. The crop system evaluated in this study represents a widespread situation in the tropics, therefore, our results can be of broad significance. Our findings suggest that traditional agriculture may not homogenize biological communities, but instead could maintain the regional pool of species through high beta diversity.

## Introduction

Agroecologists propose that traditional farming systems may provide invaluable agroecological principles needed to develop global sustainable agriculture [1]. These principles are useful in developing strategies for pest management in crops, as well as, for conservation of biodiversity in human-modified landscapes. Indeed, traditional agriculture sustains a huge diversity of organisms that in some cases may be comparable to that of natural ecosystems [2]. This diversity may benefit agroecosystems through positive effects on ecosystem functioning that increase adaptability to extreme climatic conditions, and resilience to biotic and abiotic stress [3]. However, underestimation of traditional knowledge, intensification of small family farming [4], and rural-urban migration [5] are leading to the abandonment of traditional agriculture. The combination of these factors results in an irreparable loss of native varieties of crops, their wild relatives, and associated biota that perform essential ecological functions for agriculture (e.g., pest regulation, pollination and nitrogen fixation, etc.). Therefore, agroecologists need to elucidate how traditional farming systems prevent biodiversity loss, a key factor for achieving sustainable agriculture.

The large number of species in traditional crop systems could result from high habitat heterogeneity, especially if the species exploit resources in different habitats in the landscape mosaics [6]. In addition, cultural practices for the control of pests and weeds could contribute to the maintenance of overall diversity. For instance, weeds within crops have been related to better pest suppression because natural enemies will increase in diversified agroecosystems and thereby control herbivores more effectively [7]. Furthermore, weeds could encourage longevity and fecundity of natural enemies of pests by providing them with extra resources, such as nectar, pollen and habitats [8–13]. Alternatively, however, resources provided by plants may also benefit herbivores in crops [14]; thus, increased plant diversity *per se* may not necessarily result in better pest suppression [15, 16]. Much research is still needed on the biodiversity of traditional crops and the roles of those species.

Assessments of biodiversity in human-dominated landscapes indicate that less-intensive land use has a positive effect on biodiversity [6, 17]. For example, comparison of biodiversity between organic and conventional farms has demonstrated higher species richness of weeds and beneficial arthropods in organic fields [12, 18–21]. At the landscape level, the presence of natural habitats may favor the species richness and abundance of beneficial arthropods in crops [21–24]. Although these findings are consistent across different regions and crops [25], the methodological approaches of these studies have been criticized because they have mainly focused on diversity at the plot scale [17, 26]. Consequently, the impacts of human activities on biodiversity in agricultural landscapes should consider different spatial scales in order to gain more insight into the relationships between landscape structure, crop management practices, and biodiversity.

A growing number of studies have partitioned diversity into components (alpha, beta and gamma diversity) to assess the effects of crop management practices and landscape structure on biodiversity in agroecosystems [17, 26–29]. The findings of these studies have led agroecologists to suggest that intensifying agriculture (through the transformation of natural habitats to agriculture, or through crop management practices to increase crop yields) may homogenize biological communities across agricultural landscapes [17, 28]. This homogenization may arise either from the reduced availability of niches in simplified habitats, which can promote the loss of rare or habitat specialist species, or from the dominance of disturbance-adapted species that can dominate biological communities [30]. However, agricultural intensification may also differentiate communities across agricultural landscapes; in this case, we would expect beta diversity to increase as land use intensifies. For instance, a higher contribution of beta diversity to overall diversity has been observed for weeds [27] and arthropods [26] in temperate agroecosystems.

This type of differentiation may occur if the landscape configuration restricts the dispersion of organisms across different habitats. For example, food production activities result in habitat patchiness in the landscape, which in turn promotes differentiation of communities through extinction-colonization dynamics [31]. These processes are highly dependent on the spatial scale and the organisms’ life history traits, although geographical distances between plots and similarities in environmental conditions might also be important predictors of changes in the composition of species [31]. Few studies, however, have analyzed these relationships considering multiple taxa, and instead have focused on one particular group of organisms [17, 22, 32–34].

In this article, our aim was to study plant and arthropod biodiversity in traditional cornfields located in a mountainous environment in the Colombian Andes. We analyzed diversity data with a multi-taxonomic focus (plants, herbivores and predators) and multi-scale perspective (within fields and among fields). Specifically, we addressed the following questions: *i)* How does alpha diversity change across a gradient of forest cover for different taxa in this area? *ii)* To what extent does beta diversity change among different taxa and among rare and common species? *iii)* Are environmental factors related to differences in species composition among cornfields? *iv)* How do species composition and the relative abundance of species vary among cornfields?

The presence of natural habitats in agroecosystems is associated with higher species richness of arthropods in crops [25]; therefore, we expected that plant and arthropod biodiversity in cornfields would increase with increasing forest cover surrounding the crop. The long land use history and traditional knowledge in this region—indigenous people inhabited this area before the Spanish conquest in the sixteenth century [35]—creates highly heterogeneous landscape mosaics, so we hypothesized that beta diversity could be high among cornfields. We also expected that the geographical distance between cornfields would cause species replacement, leading to higher beta diversity as the distance between cornfields increased.

## Methods

### Study area

Fieldwork was conducted from August 2011 to February 2012 in the municipality of Topaipí, Cundinamarca, a rural area located on the west slope of the Central Cordillera in Colombian Andes (5 23.366N, 74 18.125W). In this region, we chose twelve traditional cornfields with a minimum distance of 230 m from each other. All fields were small, varying in area from 591 to 5112 m^2^. Cornfields were embedded in a landscape sector of 19 km^2^ in an altitudinal gradient ranging from 1296 to 1550 m.

The landscape in this region is a mosaic of native forest and human land use, such as cultivated fields, fallows and pastures, with a forest cover of not more than 30% of the municipality [36]. Annual rainfall in the region is 2525.8 mm, with peaks in April and September, and the average temperature is 21.3°C, with a range of 19.9 to 23.3°C.

### Crop management

Although corn is a semiannual crop, farmers prefer to sow it only in the second season of the year to avoid pest problems. Therefore, land is prepared for sowing in mid-July to mid-August, primarily by slash-and-burn. This sowing after mid-August means that the emergence of corn seedlings matches the onset of rains in the beginning of September. Farmers also perform hand weeding 7–8 weeks after corn emergence, and they do not use chemical control for insect pests. The peasants partially harvest corncobs in November, but they allow the crop to dry until February or March, when the cornfield is harvested. Most of the crop biomass remains in the weedy field until the next crop season. In most cases, this cycle is repeated for 3 or 4 years, followed by a fallow period of variable length.

### Land use types surrounding cornfields and other environmental descriptors

We used GPS to record the geographical coordinates, the altitude and the area for each cornfield. We mapped the land-use types at multiple radii around each cornfield (100, 250, 500, 750, and 1000 m) via field visits and inspections of aerial photographs (Ground Sample Distance = 27.7 cm), and we estimated the amount of area covered by each land-use type using ArcView 3.2 [37]. We classified land-use types in the study area as native forest, secondary growth, hedges, pastures, transitional crops, perennial crops, home gardens, and constructions.

Land use cover surrounding each crop was selected as the main predictors for further analysis. In particular, we used the cover of native forest, natural and semi-natural habitats (native forests, hedges, and secondary growth combined), and the cover of pastures and crops as additional predictor variables.

We evaluated possible co-varying effects of soil quality on measurements of diversity by sampling soil in each plot at harvest time. The introduction of organic matter into soils may increase biomass and species numbers [38, 39], so we chose the percentage of carbon in soils as a predictor of species richness for further analysis. We also included altitude and the perimeter-to-area ratio of each field as co-variables.

Previous use of the plots differed among cornfields. Therefore, at the beginning of the study, we recorded the known former type of cover of each field. Five categories were established: native forest, fallow (secondary growth), pasture, other crops, and invaded plots (fallow plots dominated by a unique plant species).

### Arthropod sampling

We sampled flying and leaf-dwelling arthropods in September and December 2011. Samples were obtained by sweep netting (N = 10 strikes) at the center of each cornfield. Arthropods were preserved in 70% alcohol until further identification at the family level and morphospecies of herbivores and predators. We classified arthropods into trophic groups (predators, parasitoids, herbivores) according to literature records for families or genera [40–44].

### Weed sampling

We sampled plants in December 2011, four months after the corn sowing date. We randomly selected five rows in the center of each cornfield. For each row, we used equidistant sampling stations along a 20-meter-long transect, for a total of 25 sampling stations per cornfield. Each station was sampled using a 50 cm x 50 cm quadrat divided into 100 subquadrats. We recorded the presence of all plant species in each station and counted the number of subquadrats occupied by each species as a measure of cover.

A measure of dominance of each species per cornfield was estimated as the sum of their relative values of frequency and coverage. The relative frequency was estimated as the proportion of quadrats in which a species was present in each cornfield; relative coverage was the sum of the coverage of each species in all quadrants divided by the sum of the values of coverage for all species in each cornfield.

### Ethics Statement

Fieldwork and sample collection were performed within cornfields and were restricted to agricultural fauna and flora on privately owned land. The twelve landowners in the study area issued all necessary working permits.

### Data analysis

Sample completeness in each cornfield was evaluated as the percentage of species observed relative to the number of species predicted by the Abundance Coverage-based Estimator of species richness (ACE) using EstimateS ver. 8.2 [45]. In addition, we estimated the sampling coverage of our data; these values representing the proportion of the total number of individuals in a community that belong to the species represented in a sample [46].

#### Local diversity

We calculated the Hill numbers or “true diversities” of each cornfield by following the methodological approach developed by Jost [47]. According to that method, common diversity indices are converted to measures of diversity in the community, which are known as the “effective number of species” and which obey the duplication principle [47]. We calculated these numbers at three different orders (*q*) of diversity. Order *q* indicates the measurement’s sensitivity to common and rare species. A *q* value of 0 indicates indifference to species abundance, such that all species are given the same weight, thereby favoring rare species. When *q* = 1, species are weighted exactly for their abundance in the community; rare or common species are not favored, whereas *q* = 2 favors more abundant species [47]. Accordingly, species richness is a measure of diversity of order zero (^0^ *D*), the exponential of Shannon’s index is the measure of diversity of order one (^1^ *D*), and the inverse of Simpson’s index is a measure of order two (^2^ *D*) [47, 48]. We constructed diversity profiles by plotting diversities at different orders in an increasing manner, which allowed us to identify patterns of dominance in cornfield communities. True diversities were calculated using R and a modified version of the Entropy calculator, an Excel code developed by L. Jost.

We used linear regression models to examine the relationships between diversity of plants or arthropods and land use types around the cornfields, including native forest, natural and semi-natural habitats combined, crops and pastures. We constructed alternative models with land use types at different distances around the crops (100, 250, 500, 750, and 1000 m) to identify the spatial scale in which models have a better fit with observed data. To select the best models we used Taylor diagrams for comparing non-nested regression models, based on the correlation between fitted values from the models and observed values, and also the Root Mean Square (S1 Fig) [49]. The land use data over a 100 m radius around the crops showed an increasing overlap (6, 24, 38, and 51% at 250, 500, 750, and 1000 m, respectively), so we tested model residuals for spatial autocorrelation in order to ensure spatial independence between the twelve study sites [50]. We found no evidence for spatial autocorrelation at any spatial scale considered (S1 Table).

The natural variation between fields led us to use soil organic matter, altitude, and field perimeter-area ratio in the models as covariates. Before performing regression models all predictor variables were log-transformed (Ln Variable = ln (variable-(minimum (variable)-1)), to account for non-linear relationships among variables. We then controlled possible multicollinearity among predictors by calculating the Variance Inflation Factor (VIF), and included variables with VIF equal to or lower than 10 in the models [51]. Finally, we used stepwise forward and backward simplification for model specification in order to comply with the principle of parsimony and we chose the best models based on the criteria of Akaike [52]. We checked all model assumptions including homoscedasticity, normality and independence, and we only present significant models (P< 0.05) that accomplish all assumptions. All analyses were conducted using R software [53].

Additional linear regression models were performed to examine the relationship between plant and arthropod diversity. We followed the same procedures described above to avoid multicollinearity, for transformation of variables, and for model specification and simplification.

#### Turnover of species between cornfields

We used multiplicative diversity partitioning of Hill numbers in their unweighted form to analyze the changes in species composition between cornfields [47]. This method partitions the regional gamma diversity (γ) into independent components of local alpha diversity (α) and beta diversity (β) in a multiplicative manner: Dα × Dβ = Dγ [47]. Beta diversity can be transformed into compositional similarity (CS) as follows:

(^q)CS=(1/(^q)Dβ−1/N)/(1−1/N) (1)

Values of compositional similarity vary between 0 (all sampling units have different species) and 1 (all sampling units have the same species). When *q* = 0 and N = 2, the value is equivalent to the Jaccard Index; when *q* = 2, the result is the Morisita-Horn Index. Such transformation is useful when comparing values based on a different number of sampling units [48].

We performed a Mantel test to evaluate whether the variation in the pairwise beta diversity of cornfields was related to pairwise crop distance. In addition, we performed a Mantel test between the pairwise beta diversity matrices and the environmental distance matrices to determine if the dissimilarity in species composition was related to environmental gradients. Environmental distance matrices were constructed based on pairwise differences between cornfields, including forest cover surrounding each cornfield, altitude, percentage of soil organic matter and the raw number of plant species in each cornfield. Correlation coefficients and p-values were estimated from 1000 permutations. Pairwise beta diversity matrices and the Mantel Test were performed in R [53].

#### Patterns of relative abundance

For each cornfield, we ranked species according to their abundance from highest to lowest. We plotted the abundance of the ten most dominant species in each cornfield. These graphs allowed us to identify changes in the dominance and composition of the more abundant species among the cornfields.

#### Context-dependent changes in species composition

We performed Non-Multidimensional Scaling analysis to visualize changes in species compositions as a function of the land use context of the cornfield. We classified cornfields into two categories of land use contexts based on the percentage of native forest in a radius of 250 m around the crop. The first group, called the “Agricultural Context” (fields A1-A6), consisted of fields with 1 to 26% of native forest. The second group, the “Forest Context” (fields F1-F6), comprised of fields with 27–50% of native forest around the crop.

Ordination was undertaken for quantitative data using the Jaccard and Morisita-Horn Indices, which are also direct transformations of beta diversity of orders 0 and 2, respectively. The stress values of each analysis are reported in the results. These analyses were performed in R [53].

## Results

We collected 198 morpho-species of plants from 53 families; 5975 individuals of herbivores from 5 orders, 38 families and 217 morpho-species; and 1574 individuals of predators from 7 orders, 34 families and 132 morpho-species. According to the ACE richness estimator, we sampled 83% and 70% of the total estimated number of plant and arthropod species in the community, respectively. However, when we used sampling coverage values, we obtained values of 90% to 95%, suggesting that only 5% to 10% of individuals of the community belonged to species not represented in our sampling. Therefore, we conclude that our sampling was satisfactory for characterization of plant and arthropod communities within the cornfields.

### Local diversity

The alpha diversity profiles indicated a large decrease in the effective number of species—or true diversities—as the order of diversity (*q*) increased, indicating a high degree of dominance in the community (although highly variable among cornfields); thus, most of species richness was due to rare species (S2 Fig). This pattern of dominance was consistent throughout all cornfields and all taxonomic groups. Furthermore, high dominance of communities of plants and arthropods within the cornfields occurred, regardless of whether the field was mainly surrounded by agricultural land or by native forest.

Forest and agricultural cover had significant positive regression weights on models that examined the relationships between plant richness and land use covers, indicating that cornfields with larger proportion of these landscape elements were expected to have higher plant species richness (^0^ *D*), for the 500 and 750 m landscape buffers. Other land uses, such as pastures (at a spatial scale of 1000 m) had a positive relationship with the diversity of common plant species (^1^ *D*). In contrast, the diversity of abundant plant species (^2^ *D*) was not associated with any land use or environmental predictor. Altitude and soil organic matter did not contribute to regression models examining relationships with plant diversity. Finally, field perimeter-to-area ratio had a significant and positive relationship with diversity of common plant species (^1^ *D*) but only for the 1000 m landscape buffer (Table 1).

**Table 1:** Results of linear regression models for biodiversity of plants and arthropods in twelve traditional cornfields located in the Colombian Andes. Coefficient values (b) of predictor variables and P-values are shown for each model. Alternative models were constructed using land use types measured at 100, 250, 500, 750 and 1000 m. Only significant models at 0.05 confidence level are included here.

Response variable	Landscape Radius (m)	Forest		Agricultural land		Pasture		Altitude (log)		Soil organic matter (log)		Field Perimeter-to-area ratio (log)		Model fit		
		b	P-value	b	P-value	b	P-value	b	P-value	B	P-value	b	P-value	Adj. R^2^	F (d.f.)	P-value
Plant richness ^0^ *D*	500	179.75	0.017	263.46	0.008		NS		NS		NS		NS	0.61	4.51 (5, 6)	0.047
	750	141.65	0.004	367.61	0.001		NS		NS		NS		NS	0.67	12.03 (2, 9)	0.003
Plant diversity ^1^ *D*	1000	191.55	0.016		NS	367.6	0.001		NS		NS	140.31	0.048	0.64	4.96 (5, 6)	0.038
Herbivore richness ^0^ *D*	750		NS	242.94	0.047		NS		NS		NS		NS	0.27	5.08 (1, 10)	0.047
Herbivore diversity ^1^ *D*	100		NS		NS	120.1	0.0007	9.35	0.002	-9.4	0.026	173.68	0.014	0.78	8.72 (5, 6)	0.010
	250		NS		NS	89.99	0.002	3.70	0.015		NS		NS	0.67	8.37 (3, 8)	0.007
	750		NS	182.35	0.018		NS		NS		NS		NS	0.39	8.07 (1, 10)	0.018
Herbivore diversity ^2^ *D*	100		NS		NS	65.75	0.0007	4.69	0.003	-4.45	0.046		NS	0.79	9.22 (5, 6)	0.008
	250		NS		NS	50.36	0.001	2.02	0.012		NS		NS	0.72	10.21 (3, 8)	0.004
	750		NS	117.56	0.004		NS		NS		NS		NS	0.54	14.03 (1,10)	0.004
Predator diversity ^1^D	100		NS	-54.01	0.003	33.85	0.019		NS		NS		NS	0.60	5.14 (2, 8)	0.030

Forest cover did not contribute to regression models for herbivores. Instead, pasture cover (log-transformed) measured at small spatial scales (100 and 250 m) had significant positive regression weights, indicating that cornfields with a larger proportion of pastures were expected to have more diverse herbivore communities (^1^ *D* and ^2^ *D*). In addition, the cover of agriculture also had a positive relationship with the diversity of herbivore communities (^0^ *D*, ^1^ *D*, ^2^ *D*), but only when land use cover was measured at 750 m around the crop. Altitude, field perimeter-to-area ratio, and soil organic matter had significant weights in regression models for herbivore diversity; however, these relationships were not consistent at all spatial scales considered (Table 1). For instance, altitude (log-transformed) had a positive relationship with herbivore diversity (^1^ *D* and ^2^ *D*) when regression models included landscape data at 100 and 250 m. In contrast, the soil organic matter (log-transformed) had a negative relationship with herbivore diversity (^1^ *D* and ^2^ *D*), but only when models included land uses at 100 m around the crop. Similarly, field perimeter-to-area ratio had a positive relationship with the diversity of common herbivores (^1^ *D*), but only when models considered landscape types at 100 m around the cornfield.

Forest cover did not contribute to the multiple regression models examining the richness of predators (^0^ *D*) or the diversity of common or abundant predators (^1^ *D* and ^2^ *D*) in cornfields. However, agricultural cover at small spatial scales (100 m) had a significant negative regression weight, indicating that cornfields with a larger proportion of agricultural lands were expected to have lower diversity of common predator species (^1^ *D*). On the contrary, the cover of pastures at 100 m around the crop (log-transformed) had significant positive weights indicating that cornfields with a larger proportion of pastures around them had more diverse communities of common predator species (^1^ *D*). Altitude, soil organic matter and field perimeter-to-area ratio did not contribute to the regression models for predator diversity (Table 1).

**Fig 1:**
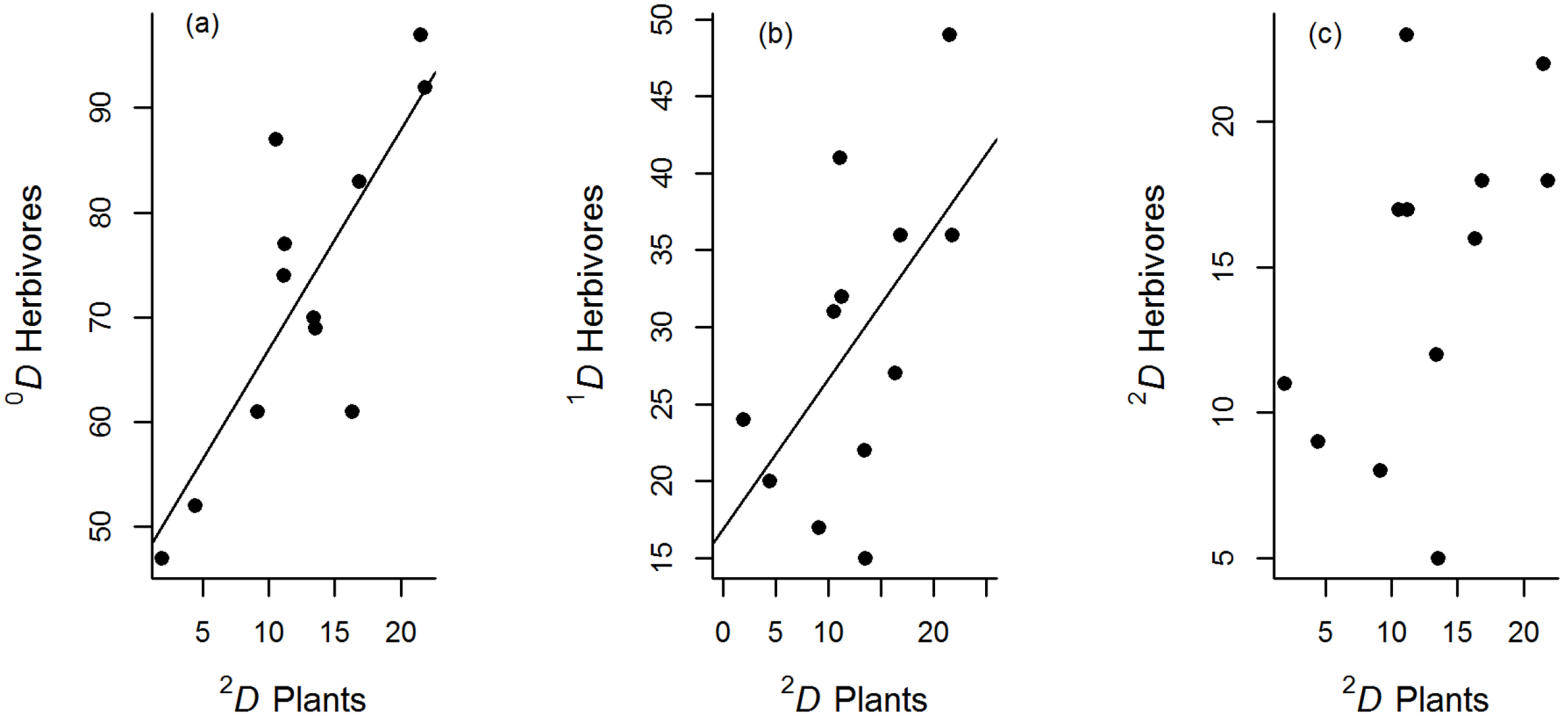
Relationships between the diversity of herbivores and plant diversity of order q = 2 in cornfields. *a)* For all herbivore species ^0^ *D*, *b)* for common herbivore species ^1^ *D*, and c) for abundant herbivore species ^2^ *D*. The plots indicate a positive relationship between the effective number of common plant species (^1^ *D*) and all orders of herbivore diversity. Lines show fitted values from significant linear regression models.

We observed a strong positive relationship between herbivore diversity (^0^ *D* and ^1^ *D*) and the diversity of abundant plant species (^2^ *D*), but this relationship was not detected for ^2^ *D* of herbivores (Fig 1 and Table 2). Similarly, plant species richness ^0^ *D* (log-transformed) had a positive relationship with predator species richness ^0^ *D*, but not with ^1^ *D* and ^2^ *D* predator diversity (Fig 1 and Table 2).

**Table 2:** Results of linear regression models for biodiversity of arthropods and plant diversity in twelve traditional cornfields located in the Colombian Andes. Coefficient values of predictor variables (b) and P-values are shown for all models. Only significant models at a 0.05 confidence level are included here.

Response variable	Plant richness ^0^ *D*		Plant richness ^0^ *D* (log)		Plant diversity ^2^ *D*		Model fit		
	b	P-value	b	P-value	b	P-value	Adj. R^2^	F (d.f.)	P-value
Herbivore richness ^0^ *D*		NS		NS	1.54	0.019	0.68	12.84 (1, 10)	0.002
Herbivore diversity ^1^ *D*		NS		NS	0.97	0.049	0.27	4.98 (1,10)	0.049
Predator richness ^0^ *D*		NS	4.47	0.042		NS	0.28	5.41 (1, 10)	0.042

Former land use seemed to affect plant diversity regardless of the order of diversity considered (Table 3). In particular, the lowest values of plant diversity were observed in fields that, prior to being sown with corn, were invaded by a dominant plant (e.g., *Hedychium coronarium* Koening or *Gynerium sagitatum* Aubl.). Similarly, dominant and common species of predators also had the lowest values of diversity in these invaded plots (Table 3).

**Table 3:** Effective number of plant and predator species (Mean and SD) according to previous land use of the cornfields.

Group	Order of diversity	Forest (n = 1)	Crops (n = 2)	Pastures (n = 2)	Fallow (n = 5)	Invaded plots (n = 2)
Plants	^0^ *D*	70.0	60.0 (2.8)	54.0 (2.8)	52.0 (13.7)	32.5 (7.8)
	^1^ *D*	27.0	26.0 (7.1)	22.0 (2.8)	20.8 (6.8)	6.5 (5.0)
	^2^ *D*	17.0	18.0 (3.5)	13.5 (4.8)	12.8 (4.8)	3.0 (1.4)
Herbivores	^0^ *D*	83.0	80.5 (16.3)	67.5 (9.2)	78.4 (14.1)	49.5 (3.5)
	^1^ *D*	36.0	25.5 (14.9)	34.0 (9.9)	30.2 (12.2)	22.0 (2.8)
	^2^ *D*	18.0	11.5 (9.2)	19.5 (4.9)	15.2 (5.4)	10.0 (1.4)
Predators	^0^ *D*	34.0	30.0 (7.1)	31.5 (3.5)	36.8 (7.2)	20.5 (2.1)
	^1^ *D*	21.0	12.0 (2.8)	16.5 (6.4)	17.2 (5.6)	7.5 (0.7)
	^2^ *D*	15.0	7.0 (0.0)	11.0 (4.2)	10.8 (5.6)	4.0 (1.4)

### Species turnover among cornfields

**Fig 2:**
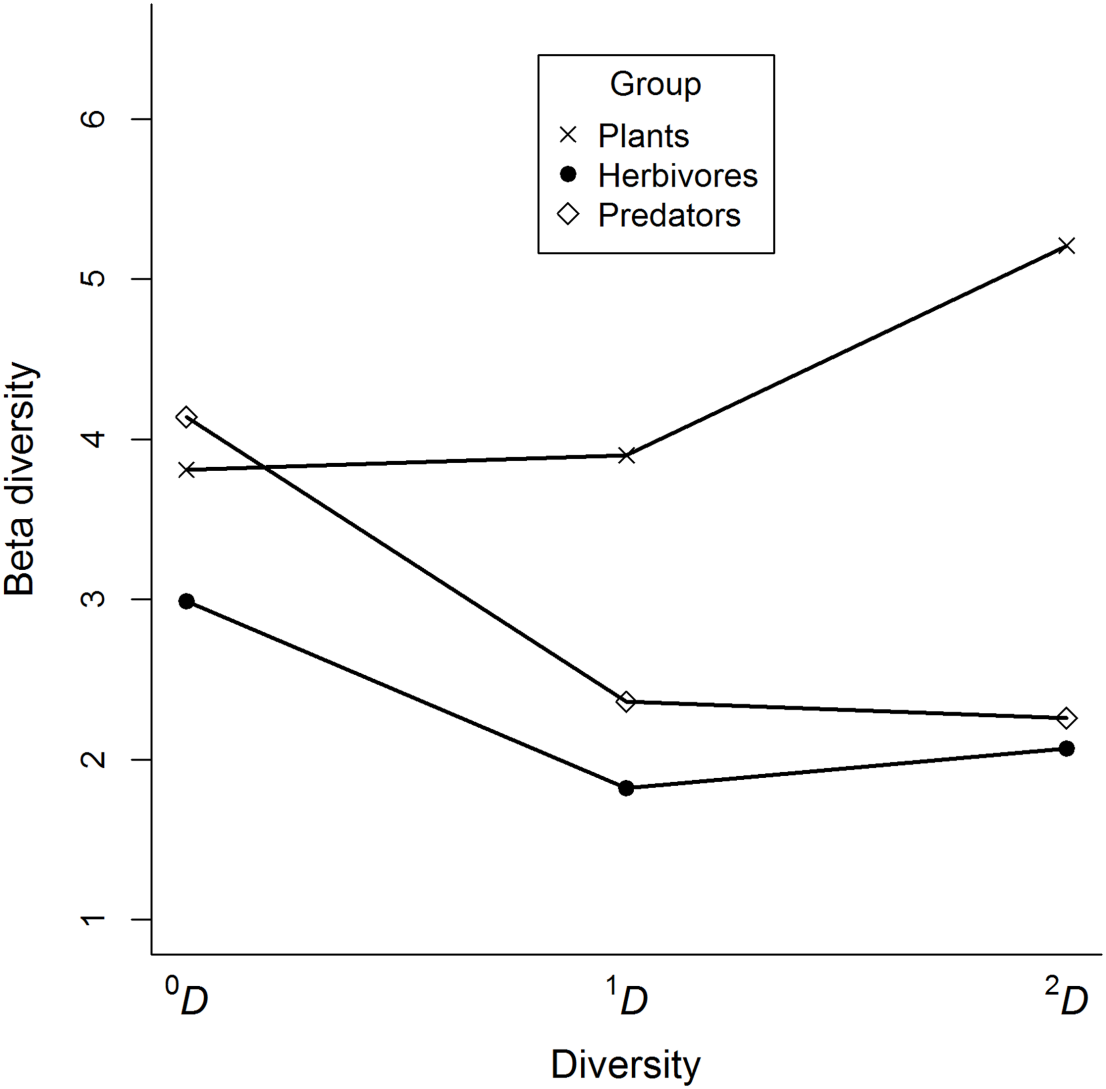
Beta diversity profiles of plants, arthropods and predators collected in twelve traditional cornfields. The plot indicates that differences in species composition among cornfields were stronger among abundant plant species, whereas for arthropods these differences arose among rare species, particularly for predators.

Beta diversity profiles indicated that the turnover of species among cornfields differed for plants and arthropods. Plant beta diversity increased as the order of diversity (q) increased, whereas arthropod beta diversity decreased (Fig 2). Consequently, the highest differences in species composition among cornfields were stronger among abundant plant species, whereas for arthropods these differences arose among rare species, and particularly among predators.

We evaluated 12 cornfields in our study area, so the true beta diversities could range in value from 1 to 12, providing an estimate of the number of effective communities in this landscape. The values for our data ranged from 1.8 to 5.2, with the lowest values for herbivores and the highest values for plant communities, regardless of the order of diversity considered (Table 4). Therefore, plant communities in this landscape tended to differ among cornfields, whereas herbivore communities tended to be more homogeneous (Table 4). Common and abundant species of predators tended to be the same for most of the cornfields, whereas rare species substantially differed among them.

**Table 4:** True diversities of plants, herbivores and predators collected in twelve traditional cornfields in an area of 19 km^2^ in the Colombian Andes.

Group	Order of diversity	Alpha	Beta	Gamma
Plants	^0^ *D*	52	3.8	198
	^1^ *D*	18	3.9	70
	^2^ *D*	8	5.2	42
Herbivores	^0^ *D*	73	2.9	217
	^1^ *D*	28	1.8	51
	^2^ *D*	12	2.1	25
Predators	^0^ *D*	32	4.1	132
	^1^ *D*	14	2.4	33
	^2^ *D*	7	2.3	16

### Spatial autocorrelation and relationships between beta diversity and environmental gradients

We found no evidence for spatial autocorrelation in the dissimilarity of species composition (pairwise beta diversity matrices) and the geographical distance between cornfields (Table 5). By contrast, the turnover of herbivores between cornfields was associated with environmental gradients in forest cover surrounding the crop and with differences in plant species richness between crop fields, in most of the spatial scales considered for land use characterization (Table 5 and S2 Table). Therefore, a greater difference in forest cover between two cornfields was associated with greater the difference in their herbivore communities (^1^ *D* and ^2^ *D*). Similarly, forest cover matrices correlated to predator beta diversity (^1^ *D*) but only when land uses were evaluated at a 250 m radius around the crop (S2 Table). Finally, our data suggest that increasing differences in the number of plant species between cornfields were associated with higher replacement of herbivore species between cornfields (Table 5).

**Table 5:** Pearsons’s r correlation for the Mantel tests between pair-wise beta diversity matrices and distance matrices for geographical location and environmental gradients in twelve traditional cornfields. Environmental gradients included differences between fields in altitude, forest cover, percentage of soil organic matter and plant species richness. Bold values indicate significant correlations at the 95% confidence level.

Group	Order of diversity	Geographical distance	Differences in altitude	Differences in forest cover (250 m radius)	Differences in soil organic matter	Differences in plant species richness
Plants	^0^ *D*	-0.04	0.15	-0.01	0.13	
	^1^ *D*	-0.19	0.07	0.00	0.18	
	^2^ *D*	-0.17	0.01	0.04	0.19	
Herbivores	^0^ *D*	0.03	-0.06	0.03	0.02	**0.33**
	^1^ *D*	0.08	0.05	**0.32**	0.21	0.05
	^2^ *D*	0.07	0.13	**0.37**	0.12	0.01
Predators	^0^ *D*	-0.21	0.01	0.23	-0.02	-0.02
	^1^ *D*	-0.16	0.02	**0.32**	0.00	0.04
	^2^ *D*	0.06	0.1	0.29	0.12	0.14

### Relative abundance patterns and changes in species composition

Rank-abundance curves for the ten most abundant species of plants, herbivores and predators confirmed that beta diversity was higher for plants than for arthropods (S3 Fig). We recorded 65 different plant species in the ten first ranks, in contrast to 34 herbivore species and 38 predator species. These graphs also revealed highly variable patterns of dominance among the cornfields. For plants, two cornfields had the highest dominance; for example, fields C5 and C7. This uneven distribution was due to the presence of invasive species such as *H*. *coronarium* and *G*. *sagittatum*, whose cover values reached up to 71% and 45%, respectively, of each cornfield. Herbivore communities also had high dominance; the highest value of relative abundance was 46%, and the four dominant species included two leafhoppers (Cicadellidae), a leaf beetle (Chrysomelidae) and a katydid (Tettigonidae). Finally, predator communities had an uneven distribution of dominant species; most abundant species included flies from the families Dolichopodidae and Empididae, spiders from the genus *Leucauge* (Tetragnatidae) and ant species from the genera *Azteca*, *Linepithema*, *Brachymyrmex* and *Ectatomma*.

**Fig 3:**
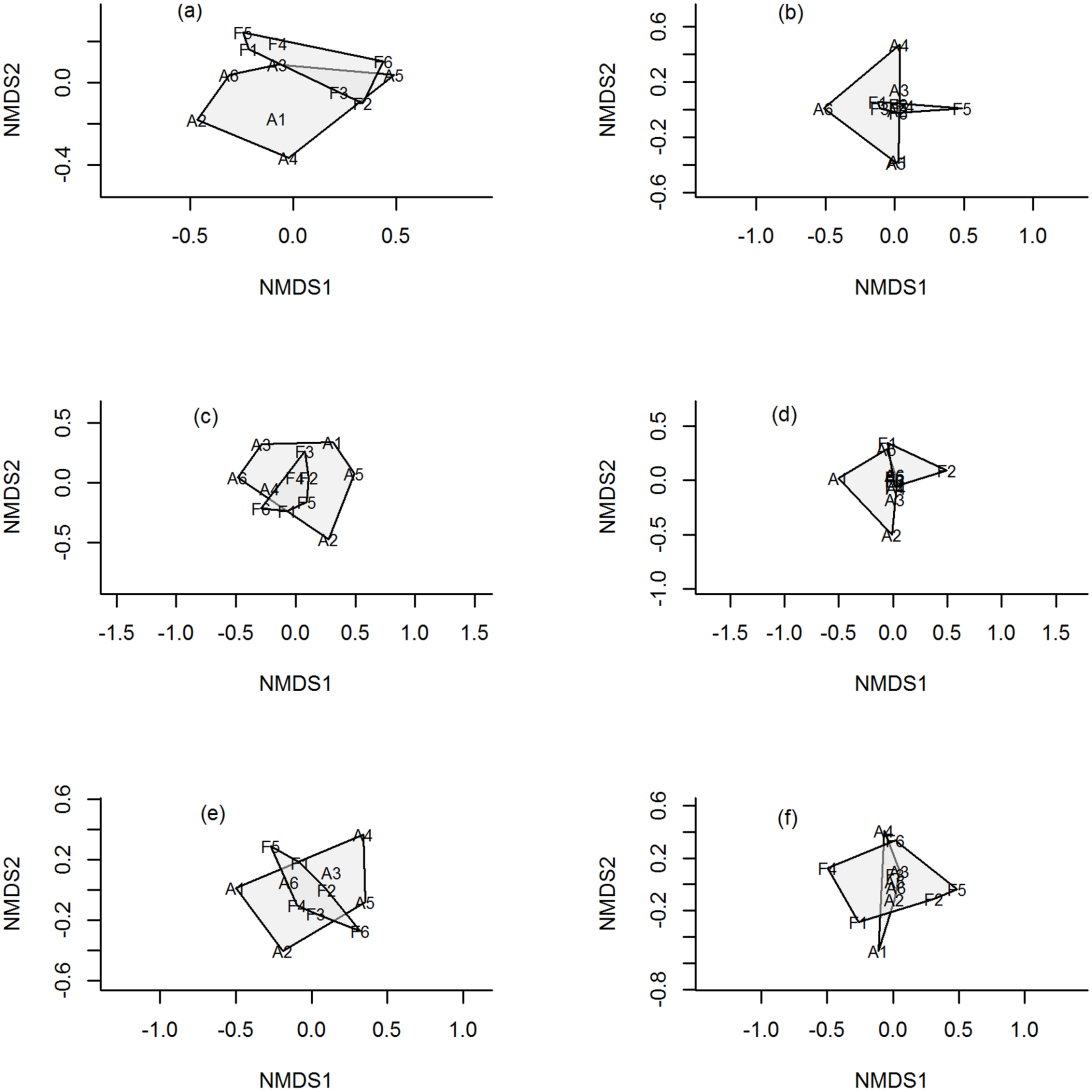
Non-metric multidimensional scaling ordination diagrams based on the Jaccard (left column) and Morisita-Horn (right column) indices for plants (a-b), herbivores (c-d), and predators (e-f) collected in twelve traditional cornfields. Ellipses indicate the clustering of biological communities based on the land use context of the cornfield: Fields A1 to A6: 1–26% of native forest in a radius of 250 m around the crop; fields F1 to F6: 27–50% of native forest around the crop. The plots indicate that dissimilarity in plant species composition was related to the land use context around the crops, however for arthropods, the plots indicate a clear overlap in species composition between land use contexts. Stress values: a = 0.01, b = 0, c = 0.16, d = 0, e = 0.18 and f = 0.

The NMDS analysis indicated that dissimilarity in plant species composition was related to the landscape context in which the cornfield was located. Ellipses joining cornfields that were mainly surrounded by agricultural habitats (A1-A6) and cornfields mainly surrounded by native forest (F1-F2) conformed two groups. This pattern was particularly evident when the similarity index was based on presence/absence data (Fig 3a), whereas some overlap occurred when the index favored abundant species (Fig 3b). For herbivore species (*q* = 0), we observed a clear overlap in the composition of species between landscape contexts (Fig 3c), whereas for abundant species (*q* = 2), there was some degree of differentiation (Fig 3d). Finally, we also observed an overlap in species predator composition between landscape contexts (Fig 3e and 3f). Therefore, the proportion of forest surrounding the crop did not have a substantial influence on the differentiation of arthropod communities among cornfields in the study area.

## Discussion

We found that diversity of plants, herbivores and predators was related to land use types around the cornfields. The positive relationship between forest cover and plant diversity suggests that cornfields with larger proportion of surrounding forest were expected to have more plant richness and plant diversity. However, plant richness also had a significant and positive relationship with the cover of agricultural lands. Therefore, forest and agricultural areas could be source habitats from where plants colonized the cornfields; supporting the notion that species could benefit from different types of habitats in the landscape [54, 55]. In addition, our characterization of land uses at different spatial scales revealed that plant communities seemed to respond more to changes in the landscape composition occurring at 500–750 m around the crop.

The central role of the forest for plant diversity was also supported by the results of NMDS analyses, suggesting that the closeness of cornfields to forests does affect the composition of plant species. For instance, the dominant species in the agricultural context were *Pteridium aquilinum* (L.), *H*. *coronarium* and *Spermacoce* sp., whereas in the forest context, the dominant species were *Impatiens balsamina* L., *Cortaderia* sp., *Brachiaria* sp., *Alocasia* sp., and *Drymaria cordata cordata* (L.) Willd. ex Schult. Other cornfield characteristics, such as the former land use could be related to diversity measurements. In particular, lower values of plant and predator diversities occurred in those fields previously invaded by a dominant plant. These results support a central role for local factors, like crop management practices in the assemblage of weed communities in crops. As discussed by Navas (2012), the structure of plant communities in agroecosystems may depend on current conditions and the legacy of previous land use, as weeds do recover from the seed bank or through vegetative reproduction facilitated by agricultural practices. We speculate that plant diversity in each field may reflect the composition and size of the seed bank, which in turn is mainly affected by field management [56]. Therefore, future research should consider the impacts of landscape configuration and field management practices on the diversity as well as the composition of the seed bank in crop fields.

Our results contrast with previous findings of a positive relationship between predator diversity and proximity to forests in agroecosystems [33, 57]. Furthermore, our data did not support the hypothesis that non-crop areas (forests and secondary growth) have a positive effect on the species richness of predators, in contrast to several previous studies with spiders [22, 23, 32]. However, we observed that diversity of common predators seemed to respond to other types of habitats in the local environment. In particular, cornfields with larger proportions of agricultural land, at small spatial scales (100 m around the crop) were expected to have lower diversity of common predator species, whereas an increased proportion of pastures enhanced predator diversity in cornfields. Similar findings of negative relationships between crop land and predator diversity have been reported previously [7]. These results suggest that predator response to landscape composition may vary among species. Thus, some predators could be affected negatively by land use intensification, whereas other species could benefit from new habitats like pastures.

The lack of a clear pattern in the response of predators to changes in land use types, may arise as a consequence of the higher landscape complexity typical of traditional agriculture landscapes. This, in turn, would enhance spillover of arthropods among different habitats types in the landscape. The moderate landscape hypothesis postulates that this spillover may have consequences in community structure by modifying the effect of landscape-wide diversity in local assemblages [6]. Thus, at least in these systems, the availability of native forest may be less important than the proportion of agricultural land or stochastic processes. Predators in cornfields may be able to obtain resources such as alternative prey, pollen and refuge in other habitats surrounding the field, including pastures and other crops. For example, a higher abundance of earwigs—a main predator of *Spodoptera frugiperda* J.E. Smith (Lepidoptera: Noctuidae) in maize crops—was found in environments with higher cover of grassland habitats in the landscape, whereas spiders and ground beetles were more abundant in environments dominated by coffee plantations [58]. Clearly, further research is needed to classify predators based on their dispersion capability, to provide a better determination of the role of the landscape composition on pest natural enemies in crop systems.

Interestingly, herbivore diversity in cornfields was not directly related to forest cover. Instead, our data suggest that cornfields with a larger proportion of crop fields (at 750m) and pastures (at 100 and 250 m) had a higher diversity of herbivores. Better models fits for herbivore diversity were obtained when land uses were characterized at 100–250 m around the crops. These results suggest that herbivore responses to landscape composition may occur at small spatial scales in this system.

Regardless of the order of diversity considered, we observed a strong and positive relationship between the diversity of plant species and herbivore diversity in cornfields. These results are consistent with the Plant Richness Hypothesis, which was initially formulated for galling insects and argues that a higher number of plant species in a given site will equate to a higher number of herbivore species [59]. Mechanisms explaining this relation include higher host specialization by herbivores and the increase in plant species *per se*, if the number of herbivore species is similar among plant species but the number of plant species per area is higher [60, 61]. Most phytophagous insect species (>70%) are specialized in their use of host plants [62], so the herbivores collected in cornfields likely exhibit a high level of host specialization.

Consistent with our expectation of high species turnover among the cornfields studied, we observed high beta diversity values, particularly for plants and predators in our study area. Dominant plant species differed among fields, which translated into high differentiation of plant communities. Predator communities also exhibited high turnover among the cornfields, but differences in composition arose among rare species. Herbivore communities tended to be more homogeneous across cornfields than plants and predators, but they also exhibited a high degree of differentiation. Our results support a high turnover of weed species in agroecosystems [63], in comparison with mobile species such as herbivores and predators [31]. Similar findings of high beta diversity values for agroecosystems have been reported for weeds [27] and arthropods [26] in temperate regions. Overall, these results indicate high heterogeneity at small spatial scales, particularly if we take into account the small size of the study area (19 km^2^). Thus, traditional agriculture could maintain or increase habitat heterogeneity, which supports a high number of species in the agroecosystem landscape. This heterogeneity may be linked to factors including complex landscape spatial configuration, and crop management practices such as weeding, polyculture, and crop rotation.

Environmental gradients typical of mountain areas may also be a factor. For instance, in mountainous environments, large changes in slope can occur at fine spatial scales, which may affect soil properties (e.g. depth, water retention capacity), which are key factors for plant development [64]. In addition, plants can modify microhabitats through facilitating or inhibiting the colonization of other species [65] and increasing habitat heterogeneity, particularly because of the high number of species inhabiting traditional cornfields. Furthermore, if we consider plants as habitats for arthropods, then plants deliver a high variety of resources and thus a higher number of potential niches, thereby increasing habitat heterogeneity, particularly for herbivores [62, 66]. This increase in habitat heterogeneity may explain why herbivore turnover was related to differences in plant richness among the cornfields.

We observed no significant correlations between beta diversity matrices and environmental distance matrices for most groups of organisms. However, for herbivore and predator communities, we observed that a higher dissimilarity in the amount of surrounding forest was associated with a higher turnover of arthropods between cornfields. This result suggests that landscape configuration—and habitat patchiness, in particular—may promote community differentiation through extinction-colonization dynamics [31]. Possible mechanisms explaining this result include a reduction in the matrix permeability to the dispersal of organisms. For example, some habitats can act as barriers to an organism’s movement, like open pastures to understory birds [67] or tall vegetation in crop borders to some wind-dispersed species such as aphids [68]. However, we found no evidence for spatial autocorrelation in our data, thus mechanisms other than matrix impermeability and limited dispersal ability of organisms should be considered as factors explaining the high turnover of species among cornfields.

Crop management practices at the local (in-field) and landscape (among fields) scales may contribute to beta diversity patterns by creating a mosaic of different disturbed patches [69]. Nonetheless, disturbances associated with crop management likely increase the productivity of the system, leading to high dominance of the fastest-growing species [70], a pattern also observed in our data. By contrast, the high turnover of rare predator species suggests that some species cannot remain in disturbed habitats, while dominant species might be agrobionts [71]. Thus, the reason why so many plant species dominate cornfields in this small sector of the landscape remains unclear. We speculate that the number of plant species might be related to the composition of the seed bank and the land-use history of the cornfields, factors that clearly warrant future research.

In summary, less-intensively used systems, such as the traditional agriculture system studied here, hold higher community dissimilarity due to larger habitat heterogeneity [8]. As a consequence, these systems maintain considerable local diversity, contributing to regional or gamma diversity. This heterogeneity might be the most important factor explaining the high plant and arthropod community differentiation in cornfields. This beta diversity is also promoted by environmental gradients in mountain areas, by high plant species richness, and by high levels of disturbance associated with agricultural practices. Thus, traditional agriculture may not homogenize biological communities; instead, it may maintain the regional pool of species through high environmental heterogeneity and beta diversity.

## Supporting Information

S1 Fig Taylor diagram displaying a statistical comparison with observations of three model estimates of plant and herbivore diversity in traditional cornfields. Alternative models used different landscape radius around the cornfields (100, 250, 500, 750, and 1000 m). In these diagrams, the observed data are indicated as the green square on the x-axis; the standard deviation of the simulated pattern is proportional to the radial distance from the origin. The external circle corresponds to correlation values between estimates and observed data, the green contours indicate the centered root-mean-square (RMS) values. Best models are those with relatively high correlation, narrower amplitude of their variations, and low RMS error (their symbols lied nearest the observed values on the x-axis). The plot indicates that models based on landscape data at 500–750 m around the crop fitted better to observed values of plant richness (^0^ *D*), whereas models based on landscape data at 100 m fitted better to observed values of herbivore diversity (^0^ *D* and ^1^ *D*).
(TIF)

S2 Fig Alpha diversity profiles (q = 0, 1, 2) of plants, herbivores and predators in 12 traditional cornfields. Farms were ordered left to right, following a gradient of native forest from 4–50%, in a radius of 250 m around the crop. The plots indicate a large decrease in the effective number of species—or true diversities—as the order of diversity (D) increased, indicating a high degree of dominance in the community (although variable among cornfields) regardless the group of organisms considered.
(TIF)

S3 Fig Rank-abundance curves for the ten most abundant species of plants, herbivores and predators collected in twelve traditional cornfields. Farms were ordered left to right following a gradient of native forest from 4–50% in a radius of 250 m around the crop.
(TIF)

S1 File Diversity data across all species per site. (XLSX)

S1 Table Results of Moran’s Test evaluating spatial autocorrelation of regression model residuals examining the relationship between biodiversity and the proportion of different land uses in twelve traditional cornfields. Land uses were measured at different spatial scales (100, 250, 500, 750 and 1000 m). No evidence of spatial autocorrelation was detected besides over a 100 m radius around the crops. Landscape data had an increasing overlap (6, 24,38, and 51% at 250, 500,750, and 1000 m, respectively).
(DOCX)

S2 Table Pearson’s r correlation from the Mantel test between Pairwise Beta Diversity matrices and distance matrices for environmental gradients in forest cover, evaluated at different spatial scales around the crops. Bold values indicate significant correlations at the 95% confidence level.
(DOCX)
