# Supplementary figures and images for: Habitat Heterogeneity Affects Plant and Arthropod Species Diversity and Turnover in Traditional Cornfields
Source: PLoS One. 2015 Jul 21;10(7):e0128950. doi: 10.1371/journal.pone.0128950 (PMC4510542; doi:10.1371/journal.pone.0128950)

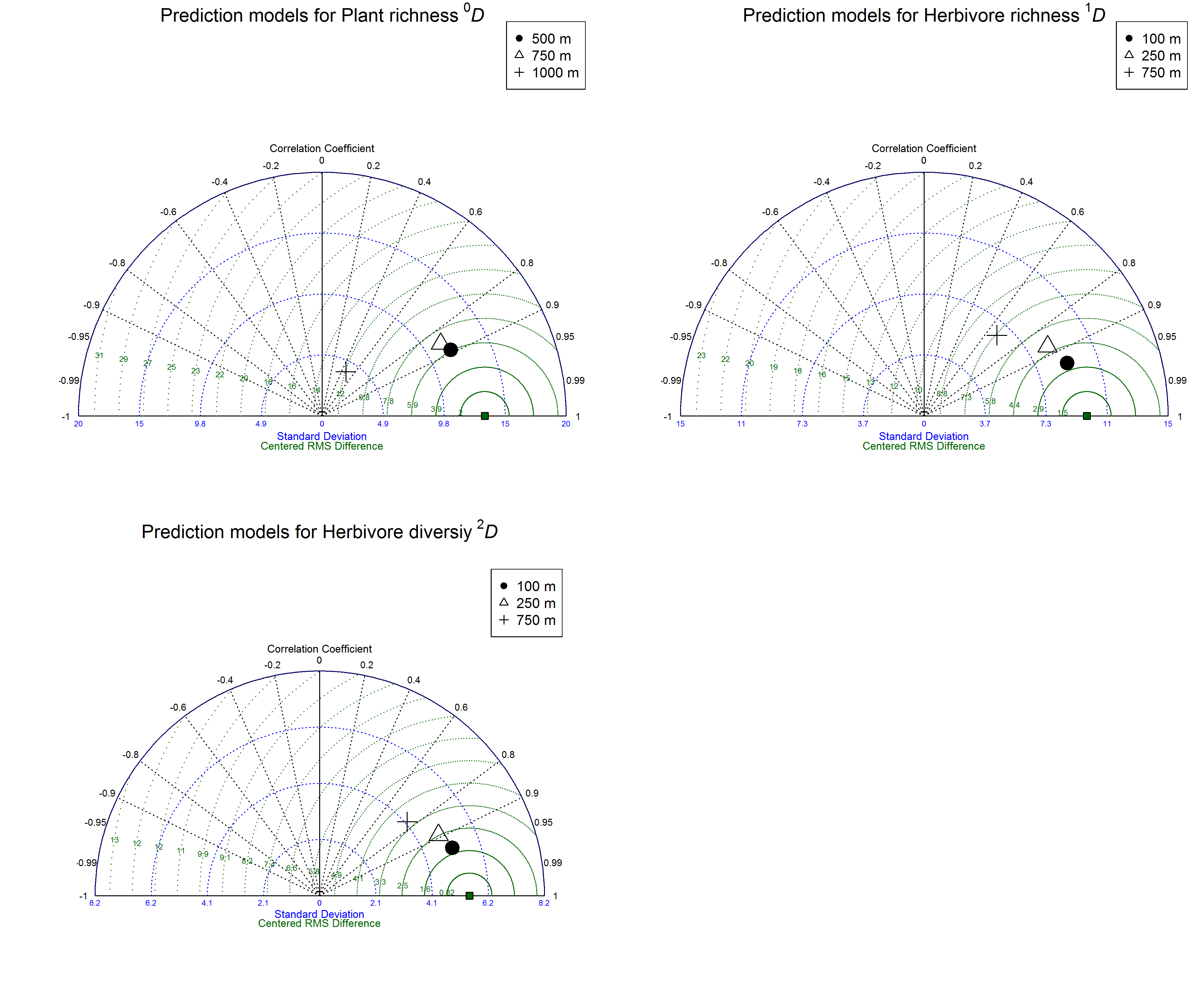

Supplement: S1 Fig — Alternative models used different landscape radius around the cornfields (100, 250, 500, 750, and 1000 m). In these diagrams, the observed data are indicated as the green square on the x-axis; the standard deviation of the simulated pattern is proportional to the radial distance from the origin. The external circle corresponds to correlation values between estimates and observed data, the green contours indicate the centered root-mean-square (RMS) values. Best models are those with relatively high correlation, narrower amplitude of their variations, and low RMS error (their symbols lied nearest the observed values on the x-axis). The plot indicates that models based on landscape data at 500–750 m around the crop fitted better to observed values of plant richness (0 D), whereas models based on landscape data at 100 m fitted better to observed values of herbivore diversity (0 D and 1 D). (TIF) [file pone.0128950.s001.tif]

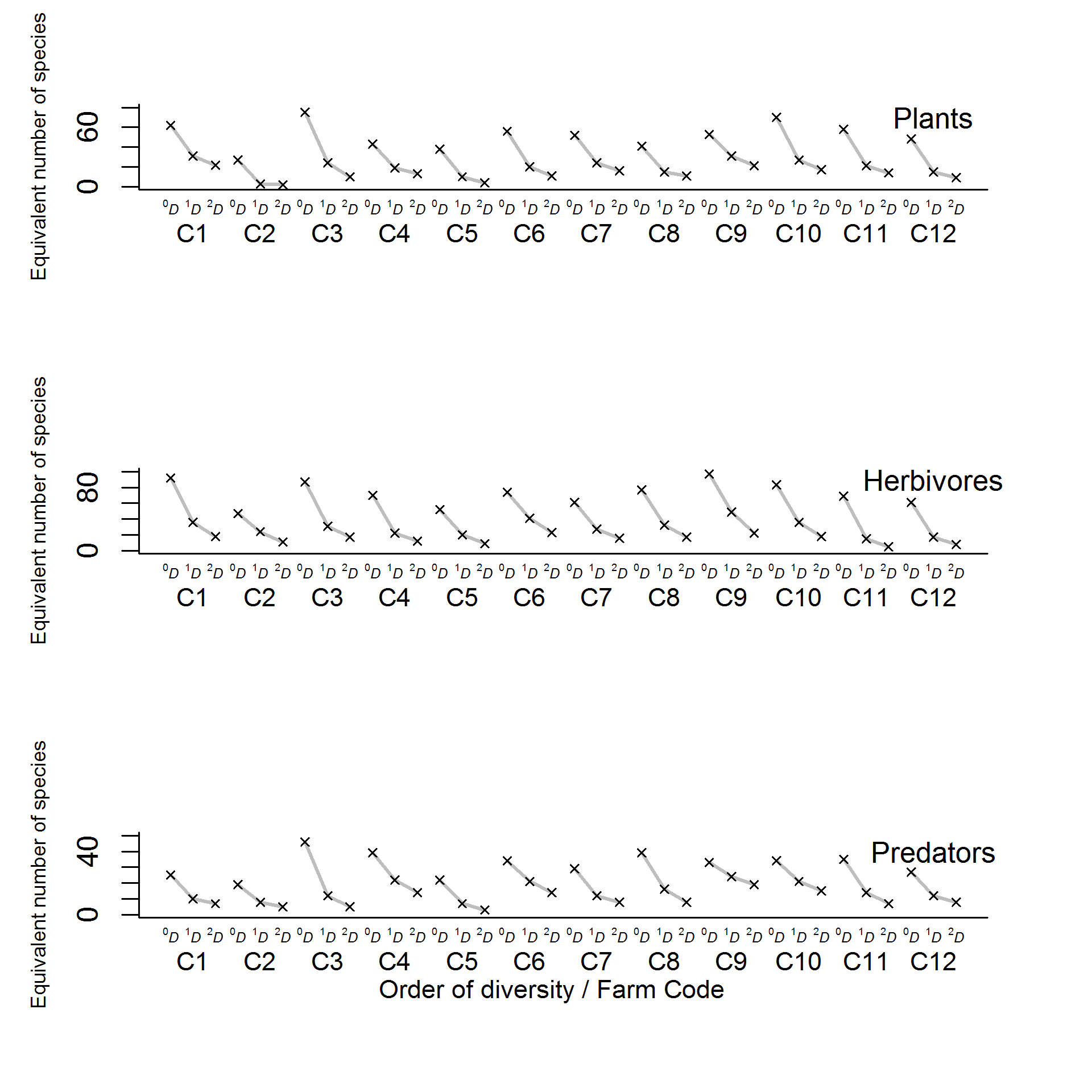

Supplement: S2 Fig — Farms were ordered left to right, following a gradient of native forest from 4–50%, in a radius of 250 m around the crop. The plots indicate a large decrease in the effective number of species—or true diversities—as the order of diversity (D) increased, indicating a high degree of dominance in the community (although variable among cornfields) regardless the group of organisms considered. (TIF) [file pone.0128950.s002.tif]

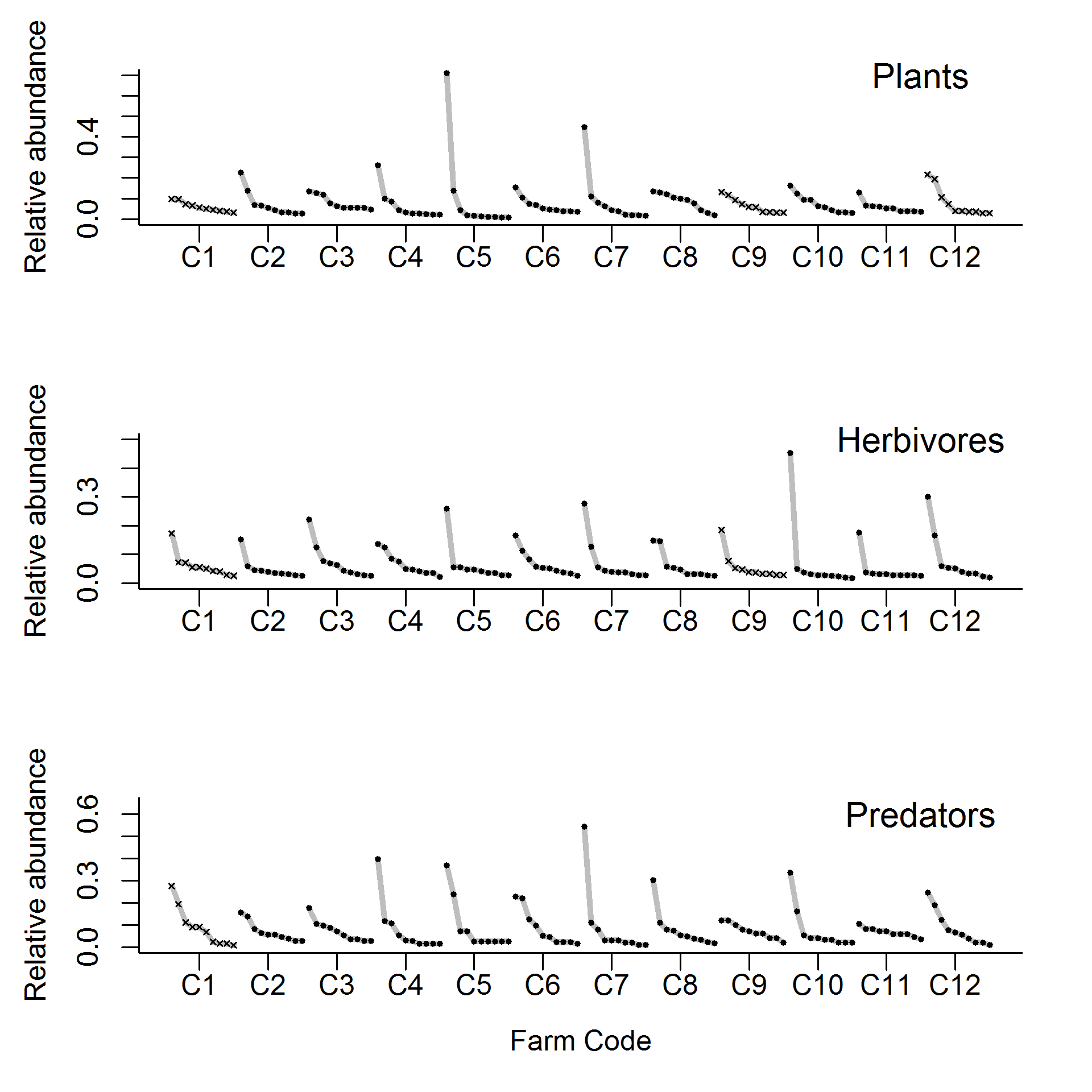

Supplement: S3 Fig — Farms were ordered left to right following a gradient of native forest from 4–50% in a radius of 250 m around the crop. (TIF) [file pone.0128950.s003.tif]
