# Supplementary material for: Habitat Heterogeneity Affects Plant and Arthropod Species Diversity and Turnover in Traditional Cornfields
Source: PLoS One. 2015 Jul 21;10(7):e0128950. doi: 10.1371/journal.pone.0128950 (PMC4510542; doi:10.1371/journal.pone.0128950)
Supplement: S1 Table — Land uses were measured at different spatial scales (100, 250, 500, 750 and 1000 m). No evidence of spatial autocorrelation was detected besides over a 100 m radius around the crops. Landscape data had an increasing overlap (6, 24,38, and 51% at 250, 500,750, and 1000 m, respectively). (DOCX) [file pone.0128950.s005.docx]

**S1 Table. Results of Moran’s Test evaluating spatial autocorrelation of regression model residuals examining the relationship between biodiversity and the proportion of different land uses in twelve traditional cornfields**.

| Response variable | Landscape radius (m) | Moran's *I* values | P-values |
| --- | --- | --- | --- |
| Plant richness ^0^*D* | 500m | -0.08 | 0.92 |
|  | 750m | -0.22 | 0.06 |
| Plant diversity ^1^*D* | 1000m | -0.18 | 0.25 |
| Herbivore richness ^0^*D* | 750m | -0.12 | 0.67 |
| Herbivore diversity ^1^*D* | 100m | -0.16 | 0.36 |
|  | 250m | -0.09 | 1.00 |
|  | 750m | -0.14 | 0.46 |
| Herbivore diversity ^2^*D* | 100m | -0.20 | 0.15 |
|  | 250m | -0.09 | 0.99 |
|  | 750m | -0.16 | 0.32 |
| Predator diversity ^1^*D* | 100m | -0.04 | 0.54 |

Land uses were measured at different spatial scales (100, 250, 500, 750 and 1000 m). No evidence of spatial autocorrelation was detected besides over a 100 m radius around the crops. Landscape data had an increasing overlap (6, 24,38, and 51% at 250, 500,750, and 1000 m, respectively)
