# Supplementary material for: Habitat Heterogeneity Affects Plant and Arthropod Species Diversity and Turnover in Traditional Cornfields
Source: PLoS One. 2015 Jul 21;10(7):e0128950. doi: 10.1371/journal.pone.0128950 (PMC4510542; doi:10.1371/journal.pone.0128950)
Supplement: S2 Table — Bold values indicate significant correlations at the 95% confidence level. (DOCX) [file pone.0128950.s006.docx]

**S2 Table. Pearson’s r correlation from the Mantel test between Pairwise Beta Diversity matrices and distance matrices for environmental gradients in forest cover, evaluated at different spatial scales around the crops**.

| Group | Order of diversity | Forest cover _(100 m)_ | Forest cover  _(250 m)_ | Forest cover _(500 m)_ | Forest cover _(750 m)_ | Forest cover _(1000 m)_ |
| --- | --- | --- | --- | --- | --- | --- |
| Plants | ^0^*D* | 0.00 | -0.01 | -0.12 | -0.04 | 0.056 |
|  | ^1^*D* | -0.01 | 0.00 | -0.09 | -0.03 | 0.083 |
|  | ^2^*D* | 0.03 | 0.04 | 0.00 | 0.01 | 0.098 |
| Herbivores | ^0^*D* | -0.05 | 0.03 | -0.18 | -0.15 | -0.046 |
|  | ^1^*D* | **0.32** | **0.32** | 0.10 | **0.25** | **0.272** |
|  | ^2^*D* | **0.37** | **0.37** | 0.14 | **0.26** | **0.274** |
| Predators | ^0^*D* | 0.07 | 0.23 | 0.20 | 0.13 | 0.035 |
|  | ^1^*D* | 0.15 | **0.32** | 0.06 | 0.10 | 0.104 |
|  | ^2^*D* | 0.19 | 0.29 | 0.00 | 0.07 | 0.083 |

Bold values indicate significant correlations at the 95% confidence level.
